# Harnessing the power of machine learning into tissue engineering: current progress and future prospects

**DOI:** 10.1093/burnst/tkae053

**Published:** 2024-12-06

**Authors:** Yiyang Wu, Xiaotong Ding, Yiwei Wang, Defang Ouyang

**Affiliations:** State Key Laboratory of Quality Research in Chinese Medicine, Institute of Chinese Medical Sciences (ICMS), University of Macau, Avenida da Universidade, Taipa, Macau SAR, 999078, China; Jiangsu Provincial Engineering Research Center of TCM External Medication Development and Application, Nanjing University of Chinese Medicine, 138 Xianlin Avenue, Nanjing, Jiangsu, 210023, PR China; School of Pharmacy, Nanjing University of Chinese Medicine, 138 Xianlin Avenue, Nanjing, Jiangsu, 210023, PR China; Jiangsu Collaborative Innovation Center of Chinese Medicinal Resources Industrialization, Nanjing University of Chinese Medicine, 138 Xianlin Avenue, Nanjing, Jiangsu, 210023, PR China; Jiangsu Provincial Engineering Research Center of TCM External Medication Development and Application, Nanjing University of Chinese Medicine, 138 Xianlin Avenue, Nanjing, Jiangsu, 210023, PR China; School of Pharmacy, Nanjing University of Chinese Medicine, 138 Xianlin Avenue, Nanjing, Jiangsu, 210023, PR China; Jiangsu Collaborative Innovation Center of Chinese Medicinal Resources Industrialization, Nanjing University of Chinese Medicine, 138 Xianlin Avenue, Nanjing, Jiangsu, 210023, PR China; State Key Laboratory of Quality Research in Chinese Medicine, Institute of Chinese Medical Sciences (ICMS), University of Macau, Avenida da Universidade, Taipa, Macau SAR, 999078, China; DPM, Faculty of Health Sciences, University of Macau, Macao SAR, China

**Keywords:** Tissue engineering, Biomaterials, Scaffolds, Artificial intelligence, Machine learning

## Abstract

Tissue engineering is a discipline based on cell biology and materials science with the primary goal of rebuilding and regenerating lost and damaged tissues and organs. Tissue engineering has developed rapidly in recent years, while scaffolds, growth factors, and stem cells have been successfully used for the reconstruction of various tissues and organs. However, time-consuming production, high cost, and unpredictable tissue growth still need to be addressed. Machine learning is an emerging interdisciplinary discipline that combines computer science and powerful data sets, with great potential to accelerate scientific discovery and enhance clinical practice. The convergence of machine learning and tissue engineering, while in its infancy, promises transformative progress. This paper will review the latest progress in the application of machine learning to tissue engineering, summarize the latest applications in biomaterials design, scaffold fabrication, tissue regeneration, and organ transplantation, and discuss the challenges and future prospects of interdisciplinary collaboration, with a view to providing scientific references for researchers to make greater progress in tissue engineering and machine learning.

Highlights

- The development and application challenges of three key elements in tissue engineering—scaffolds, growth factors, and stem cells—are reviewed.
- Supervised learning, active learning, and machine learning-based multi-objective optimization, three major machine learning methods that are expected to solve tissue engineering challenges, are discussed.
- Applications of machine learning in property prediction and optimization of biomaterials, scaffold design, and 3D printing are described.
- The challenges and prospects of combining machine learning with tissue engineering are considered.

## Background

Tissue defects and loss can be caused by congenital disease, injury, or surgical removal [1]. Although great progress has been made in organ transplantation for patients with severe tissue damage or organ failure, the insufficient supply of donor organs and long-term exposure to immunosuppressants can cause serious side effects [2]. Tissue engineering aims to mimic tissue structures or functional substitutes for the regeneration of organs. In its short history of ~40 years, this discipline has become the cradle for the birth and development of new knowledge in basic science and applied sciences [3,4]. The term ‘tissue engineering’ was not established until 1987 by the National Science Foundation of the United States, while skin grafts have been documented as far back as 3000 BC in Sanskrit. Epicel, is the first cell-based tissue engineering product which is derived from keratinocytes of the recipients for the treatment of severe skin burns [5]. Subsequently, Organogenesis (Canton, MA, USA) marketed a composite skin product for the treatment of chronic wounds, and Integra Lifesciences Inc. (Plainsboro, NJ, USA) marketed collagen- and glycosaminoglycan-based dermal matrix products for clinical promotion. In the mid-1980s, scientists prospectively proposed that suitable scaffolds should be designed to transport cells, and successfully implanted living cells into biodegradable and biocompatible polymer scaffolds as functional tissue equivalents [6]. The tissue engineering industry experienced ups and downs and gradually matured over the subsequent 40 years. With the rapid development of scaffold technology, stem cell therapy, microRNA, organoids, nanomedicine, and 3D bioprinting [7], tissue engineering has successfully enabled the creation of replacement tissues such as bone, cartilage, skin, blood vessels, and nerves. It also focuses on the development of targeted drug delivery, disease modelling, and high-throughput screening technologies [8–10]. The traditional tissue engineering strategy usually involves one-at-a-time type experiments. However, this approach proves to be time-consuming and expensive, failing to capture the intricate interplay between input variables and output results. This limitation significantly impedes the advancement and utilization of tissue engineering materials and technologies.

Artificial intelligence (AI) is a broad research field that focuses on creating intelligent agents that mimic or exceed human intelligence. Machine learning, as a crucial component of AI, provides tools and techniques for AI systems to learn from data and perform intelligent tasks. Recently, benefiting from the advancements in computation power, algorithm innovations, and large data availability, machine learning has been developing rapidly [11]. In recent years, machine learning applications in biomedical studies have increased quickly [12–14]. For example, machine learning-powered computational drug and biomaterials research and development is becoming more attractive due to its characteristic virtual screening, optimization, and design [15–18]. Moreover, machine learning models developed with medical images like magnetic resonance imaging and computerized tomography scans can efficiently enhance diagnosis in early disease detection [19,20]. Additionally, analyzing multi-modal experimental data using machine learning techniques can assist people in better simulating and understanding complex biological processes [21,22]. Over the years, machine learning-related tissue engineering research has grown rapidly, highlighting the wide range of applications and increasing the significance of machine learning within the realm of tissue engineering.

This review will first introduce research progress and current challenges in tissue engineering, and then provide an introduction of machine learning approaches that are expected to address these challenges. Subsequently, the applications of machine learning in tissue engineering will be comprehensively summarized. Finally, the review will discuss the challenges and prospects of using machine learning technology to accelerate tissue engineering research. To conclude, this review underscores the crucial potential of machine learning technologies in advancing the tissue engineering field, aiming to offer a scientific reference for researchers to apply machine learning technology in the field of tissue engineering.

## Review

### Tissue engineering

Scaffolds, growth factors, and stem cells are three key elements in tissue engineering [23,24]. Scaffolds are used to construct temporary structures/skeletons and act as the extracellular matrix for promoting cell proliferation and tissue regeneration [25]. Growth factors are well studied in the regulation of cell differentiation, proliferation, and migration [26] while facilitating tissue regeneration [27,28]. Stem cells have the potential of self-replication, multidirectional differentiation, and homing. Stem cells also have great potential in restoring the regenerative ability of functionally impaired tissues and alleviating metabolic disorders [29]. Applications of different types of scaffolds, growth factors, and stem cells have played a key role in promoting the development of regenerative medicine, but the urgent need to introduce new technologies and interdisciplinary integration to further develop the field of tissue engineering has also been exposed.

#### Scaffolds

Scaffolds are excellent materials for repairing and improving tissue function, providing a platform rich in cytokines or other functional molecules for promoting cell survival, proliferation, differentiation, and migration [30]. Scaffolds are being produced in various forms such as 3D printing scaffolds, hydrogels, fibre scaffolds, and leached scaffolds, and are extensively studied for their potential in regenerative bone, cartilage, cardiovascular, nervous system, skin, liver, teeth, and other medical applications. However, the question of what constitutes the optimal scaffold in tissue engineering and regenerative medicine remains unclear. The use of scaffolds in tissue engineering research now faces several challenges, including inappropriate mechanical properties, geometric size, porosity, surface function, biological activity, residual solvent toxicity, biocompatibility, and immunogenicity [31,32]. The current goal in the manufacturing of scaffolds is to combine cell functions with favorable biocompatibility, low-immunogenicity, and non-cytotoxicity [5,33]. However, finding a balance between the multiple requirements to optimize the tissue regeneration performance of scaffolds is challenging. Another challenge is that many of the parameters are opposite in nature, e.g. an increase in porosity that enhances mass transport also reduces the compressive strength of the scaffold [34]. Therefore, researchers urgently need new technology to help select the best combination of manufacturing parameters in a short time to predict the biological feasibility of related designs.

#### Growth factors

Growth factors are multifunctional polypeptide substances that can transmit signals between cells and regulate cell adhesion, growth, proliferation, migration, and extracellular matrix synthesis. Although growth factors are an effective means of regeneration and repair, there are still some problems in their use. For example, single release of growth factors simulates the real physiological repair process, but delivery of growth factors in a sufficient physiological dose and in the optimal proportion and sequence is difficult to control. Scientists attempt to understand the type, dose, and ratio of growth factors required at different stages of tissue regeneration. An improper combination of growth factors may trigger multiple signal cascades, resulting in negative results. Meanwhile, various growth factors are often limited to certain activities and there are still numerus growth factors whose different active functions need to be explored. At present, direct extraction and identification are commonly utilized in polypeptide development, but time-consuming, complicated steps and large functional uncertainties limit their application. For this reason, researchers and clinicians urgently require a high-tech method that can quickly and efficiently predict peptide/protein function (e.g. through structural analysis and database mining) to guide the rational use of growth factors.

#### Stem cells

Stem cells with unlimited self-renewal ability provide a variety of highly differentiated daughter cells [35]. Stem cells are widely used in the treatment of cardiovascular diseases, immune deficiencies and metabolic disorders [36], liver and kidney diseases [37], type 1 and type 2 diabetes [38], dentistry [39], wound healing [40], and immune system disorders [41]. Although stem cells have become an extremely important and almost inexhaustible resource, the technology for their application still needs to be improved. For example, isolation and purification of stem cells from the mixture cell source remains a big challenge in general practice [42], while loss of the stemness that inhibits their functions and capability to differentiate into specific cell types also continues to limit their application [33]. The current quality control of stem cell culture *in vitro* is still based on visual inspection and personal-experience judgment of researchers, which seriously hinders the standardization and large-scale manufacture of stem cell cultures. Additionally, the types and functions of stem cells are complex, and different physiological tissue structures and even different individual tissue conditions have different requirements for the use of specific stem cells. Therefore, researchers urgently need a technology that can rapidly screen stem cells that meet therapeutic criteria based on various cellular factors (such as cell shape, size, density, etc.); select the right type of stem cells with the required tissue or individual characteristics (genetic information and medical history, etc.); and even screen out the best cells in the stem cell population. At the same time, the optimal dose and cell delivery time of stem cells must be determined to help track cell migration and survival after delivery and detect any adverse reactions in order to maximize the therapeutic effects [43].

#### Challenges of tissue engineering

Creating sophisticated tissue engineering strategies is a multifaceted endeavour, requiring consideration of numerous parameters and correlations among materials, implants, manufacturing conditions, and the physiological characteristics of tissues. This process operates within a high-dimensional design space, encompassing the impacts of external chemical and mechanical stimuli, local immune responses, and other factors on tissue growth and sustainability [5,44]. For decades, therapeutic strategies of tissue engineering have been largely constrained by continuous cycles of single laboratory tests and analytical evaluations, but this process is costly, time-consuming, and inefficient, and fails to show complex correlations between input variables and related outputs, severely hampering the development and clinical translation of biomaterials and tissue engineering structures [3]. Obstacles such as intricate tissue engineering involving multiple cell types, issues like protein instability, insufficient peptide development and design, the presence of tissue-stimulating molecules, challenges in nerve rewiring, complexities related to micro-vessels, blood flow obstruction, risks associated with limb revascularization, tissue degradation, thrombosis, chromosomal instability, tumor potential, and accidents significantly hinder the widespread application of engineered tissues in biomedicine [45] (Table 1). Given these shortcomings and challenges, a high-tech method that can predict experimental data and results and reveal underlying features connecting complex data is needed. Scientists will be then advised on how to enhance the optimal design of the experimental protocol and predict the correlation between a specific treatment strategy and tissue growth potential, while being effective in terms of time and resources.

**Table 1:** Overview of common challenges in tissue engineering

Category	Challenge of experimental results prediction	Challenge of parameter optimization
Scaffold	Impossible to predict the biological feasibility of scaffold design; impossible to optimize the tissue regeneration performance of scaffolds, including biological activity, biocompatibility, and the impact of scaffolds on tissues and organs	Impossible to optimize raw materials, scaffold structure, and manufacturing technical parameters, including mechanical properties, geometry, porosity, surface function, and residual solvent toxicity
Growth factor	Impossible to predict the functional activity of proteins/peptides by structural analysis, and the development of functional protein peptides is hindered	Impossible to determine the optimal type, dosage, and proportion of growth factors required for each stage of the tissue regeneration process
Stem cell	Impossible to control the direction of stem cell differentiation	Impossible to define the quality-control criteria of stem cells cultured *in vitro*; impossible to select the appropriate type of stem cells according to the individual characteristics of patients

### Machine learning for tissue engineering challenges

**Figure 1:**
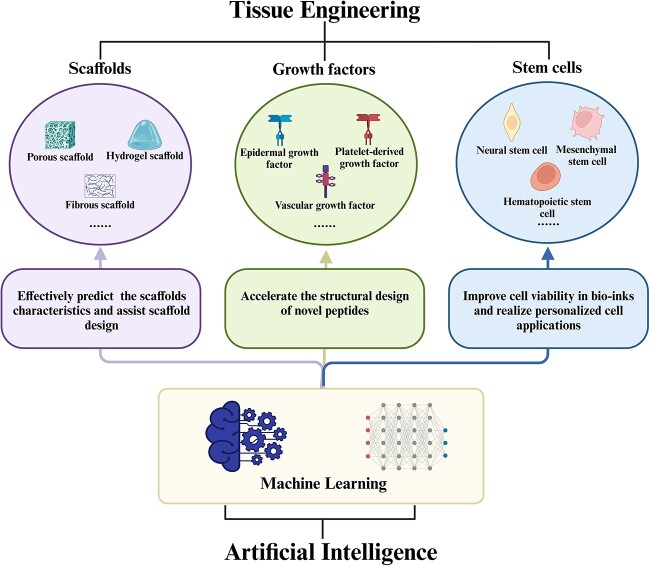
Three aspects showing how machine learning facilitates the development of tissue engineering. Created with BioRender.com

Machine learning has shown great power in tissue engineering applications. Compared with other medical research methods (laboratory and clinical research), machine learning is low-cost, fast, and efficient, and has been used to enhance and simplify materials development, scaffold design, personalized diagnosis, and treatment [3]. This review will present several machine learning paradigms and strategies that promise to solve tissue engineering challenges (Figure 1).

#### Supervised learning in tissue engineering

Supervised learning is one of the machine learning paradigms where algorithms are trained on entirely labelled training data, which promises to solve the challenge of unpredictable experimental results in tissue engineering and make the experimental process efficient. By developing machine learning models based on existing experimental results, the researcher can predict the miscibility of different new polymer blends, where the features characterizing the polymers are the input data and the miscibility (immiscible and partially miscible) is the output label [46]. Supervised learning relies on existing data to train the model to predict the output of the problem to be solved. Therefore, the accuracy of supervised learning methods is highly dependent on the selection of prior data.

Currently, supervised learning is the most used in biomedical research, which involves primarily two kinds of tasks: classification and regression. Common algorithms include classic machine learning algorithms, which mainly include linear regression, decision trees, random forests (RF), support vector machines, as well as neural networks, such as fully connected neural networks, convolutional neural networks (CNN), recurrent neural networks, and transformers, etc. By developing classification and regression models using labelled data, supervised learning provides a data-driven approach to tissue engineering research that enables more accurate and rapid analysis and prediction, such as predicting the degradation rate of scaffolds [47] and determining biological 3D printing parameters [48]. It is worth noting that the ‘no free lunch’ theorem [49] is a fundamental principle in machine learning, which states that no single algorithm is universally superior for all problems. Machine learning algorithm selection relies heavily on understanding the problem domain and the data at hand, which is usually tuned dynamically to obtain optimal performance rather than fixing a ‘superior’ machine learning method.

**Figure 2:**
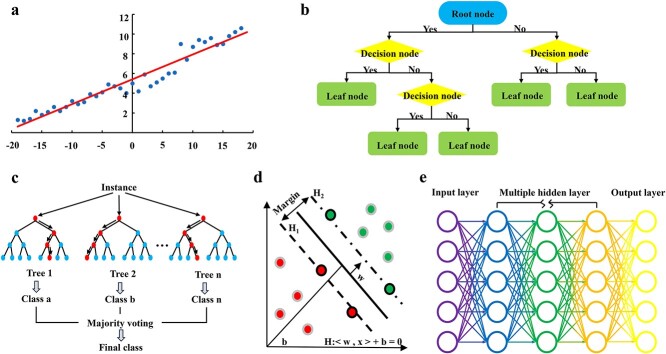
Schematic diagram of the main supervised learning algorithms: (**a**) linear regression; (**b**) decision trees; (**c**) random forests; (**d**) support vector machines; and (**e**) neural networks

Prediction models developed by various supervised learning algorithms can forecast the experimental results in advance, thereby guiding tissue engineering studies. For example, based on model predictions, researchers can prioritize materials for experimental testing or adjust the design of some experimental parameters such as pH, which can improve screening efficiency. Here we present the characteristics of commonly used supervised learning algorithms and their applications in the field of tissue engineering (Table 2) [3,50]. A schematic diagram of the supervised learning algorithm is shown in Figure 2 [51–54].

**Table 2:** Features and applications of supervised learning algorithms

Algorithm	Characteristic	Category	Applications	Benefits	Limitations
Linear regression	The linear relationship between input features and output labels is described by fitting a line or a multidimensional plane.	Regression	Material design, material biocompatibility [51]	Simple to implement and interpret	Not suitable for non-linear relationship data
Decision trees	A tree structure constructed by recursively dichotomizing the input features, where each leaf node corresponds to a classification label or regression value.	Classification regression	Cell classification, bone health assessment [52]	Easy to interpret Efficient Can handle nonlinear data	Prone to overfitting Sensitive to small changes in data
Random forests	An ensemble method, combining the predictions of multiple decision trees through bagging.	Classification regression	Comprehensive parameter evaluation [53]	High accuracy and robustness Less prone to overfitting compared to individual decision trees	Computationally intensive with large-scale data
Support vector machines	Find an optimal hyperplane that best separates different classes of samples in the input feature space.	Classification regression	Material identification and classification [52]	Effective in high-dimension data Less prone to overfitting Can handle non-linear data	Computationally intensive with large-scale data Limited interpretability
Neural networks	Mimic the computational model of the human brain by using nodes of neurons that are interconnected in a network.	Classification regression	Prediction of material properties, biocompatibility assessment of implants [52,54]	Powerful learning capability Learn features from data automatically	Complexity ‘Black box’ is hard to interpret Easy to overfit especially on small datasets

#### Active learning in tissue engineering

**Figure 3:**
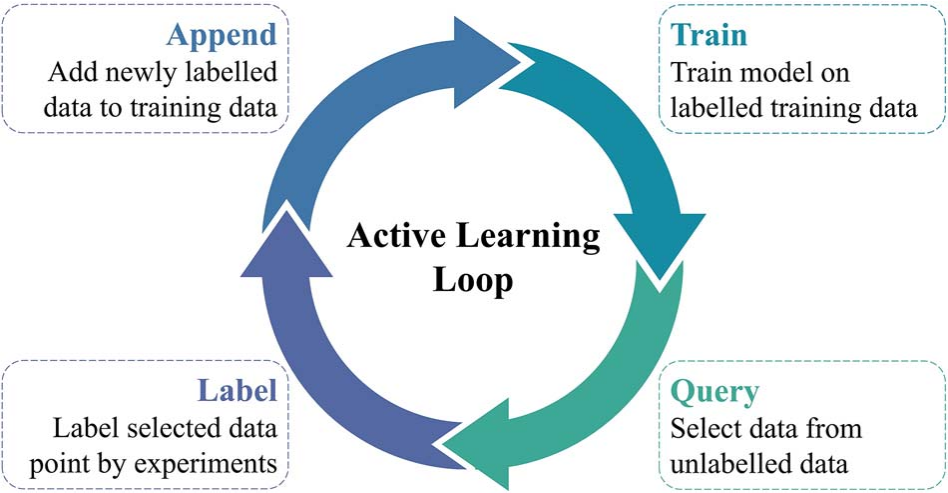
Schematic diagram of the active learning loop

In tissue engineering, datasets are often limited due to the challenges of collecting experimental data. Small datasets hinder the ability of supervised learning to generalize effectively, since when the data are not in the domain of the machine learning models, the predictions can be uncertain [55]. The adoption of active learning (AL) methodologies emerges as a promising solution in this situation. AL aims to strategically select the data points for labelling to maximize the model learning performance with minimal labelling effort. This strategy facilitates the effective interactions between machine learning models and tissue engineering experiments to achieve the purpose with limited resources. As shown in Figure 3, AL can start by using a few labelled data points to train the machine learning models (train). Then the query strategies are utilized to select data points from unlabelled data (query). Subsequently, these selected data will be labelled through experts or experiments (label). These newly labelled data points will be added to the training set (append) and used to reconstruct new machine learning models. The new machine learning models will give the new query data for the next round of labelling. This ‘train–query–label–append’ cycle will be implemented iteratively until the set metrics are met (e.g. model performance, the number of iterations, and exhausted experimental sources). AL has currently been used to address problems including chemical synthesis [56] and drug formulation development [57], and is beginning to be applied in tissue engineering [58].

AL is good at identifying the optimal choice from a number of candidates as soon as possible, which gives it great application potential in tissue engineering. In scenarios where data are difficult to obtain, such as complex scaffold design and personalized medicine strategy design, AL can help target the most likely objectives through the efficient ‘train–query–label–append’ loop beginning with just a few data points. In scenarios where search space is huge, such as large-scale biomaterial screening, the prediction models developed with limited data may not be sufficient to make accurate predictions for most data out of the models’ applicability domains. However, by updating models iteratively and efficiently, AL can rapidly broaden the model prediction boundaries, thus improving the efficiency of large-scale screening.

#### Machine learning-based multi-objective optimization

Multi-objective optimization (MOO) problems are common in tissue engineering [59–61]. Although different machine learning models can be developed to evaluate the material parameters separately, potential interactions or dependencies between parameters may be overlooked. In contrast, MOO can identify trade-offs and synergies between different parameters simultaneously, aiming to find optimal solutions that balance competing objectives across all parameters. Although traditional methods like the non-dominated sorting genetic algorithm and multi-objective evolutionary algorithms have been applied in tissue engineering MMO studies [62], machine learning-based MMO approaches have recently been more and more popular due to their efficiency, especially in complex objective spaces [60]. Bayesian optimization (BO) is one of the most commonly used strategies, which is a sequential model-based optimization technique that uses surrogate models to guide the search for the optimal solution. BO has been used in MOO of variables for neotissue growth on a 3D scaffold in a bioreactor setting, which showed better performance than the random search and a variety of genetic algorithms [59]. Besides, reinforcement-learning-based MMO is another promising approach. Reinforcement learning is inspired by how people and animals learn from the environment, working via a framework for teaching machines to make a sequence of decisions by interacting with an environment. The agent receives feedback (rewards or penalties) after performing actions to iteratively optimize decision strategies in order to maximize the long-term reward. Such a capacity to learn dynamically from trial and error makes it particularly suitable for complex tasks that involve decision-making and optimization, such as optimizing personalized drug dosages [63] and radiotherapy [64], as well as the MOO of small molecule drugs [65,66].

Machine learning-based MMO approaches can effectively help balance multiple requirements in tissue engineering research. Besides optimizing the multiple properties of materials/scaffolds simultaneously, MMO approaches can also tailor tissue engineering solutions for patients by balancing different therapeutic goals.

#### General workflow of developing machine learning models

**Figure 4:**
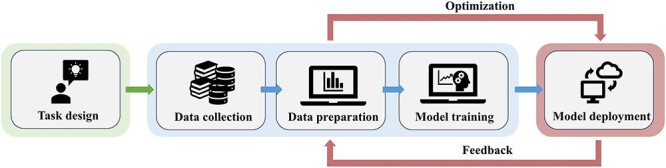
General workflow of developing machine learning models.

Developing machine learning models follows a systematic workflow (Figure 4). Task design is usually the first step, which clearly defines the specific problems that need to be addressed and the types of data that need to be collected. This is followed by data collection and preparation. Next comes model training. The data are divided into training data and validation/test data, where the models are trained on the former while the latter are used to evaluate model performance. Finally, the trained model will be deployed for real-world applications and updated as new data or advanced algorithms become available. Following this iterative and dynamic workflow of machine learning, model performance will be improved continuously, leading to increasingly accurate prediction and a broader range of applications.

### Machine learning applications in biomaterials and scaffolds

#### Machine learning in property prediction and optimization of biomaterials

Traditional methods to optimize the properties and functionalities of biomaterials often rely on iterative experimentation, which can be resource-intensive and time-consuming. The emergence of machine learning provides a new research paradigm for biomaterials. Machine learning models can more effectively predict and evaluate the properties and interactions between biomaterials and physiological systems by analyzing data from a large number of different biomaterials and manufacturing technologies [67,68], thereby optimizing the design and manufacture of material structures for specific applications and reducing the time and cost of loss in the process.

A linear regression algorithm is a kind of machine learning algorithm designed for regression tasks. It works by fitting a linear equation to figure out the relationship between the output target and input features. This method has been successfully used to determine the correlation between magnetic resonance imaging output and proteoglycan content in cartilage matrix [69] and to determine the effect of specific polymer materials on reducing bacterial attachment [51]. Neuro-fuzzy logic technology is a hybrid method that integrates fuzzy logic and neural networks to reveal the complex relationships between variables. It has been used to establish the rules of how varying compositions, properties, and concentrations affect the antibacterial effects of bioactive glasses [70]. Artificial neural networks (ANNs) typically describe shallow neural networks with just several hidden layers, which are widely used in regression and classification problems. The successful applications of ANN encompass a diverse range, including the prediction of the water contact angle, the protein absorption of self-assembled monolayers from molecular structures [71], and the identification of promising biomaterials for bone tissue engineering [52], as well as unveiling relationships between the molecular properties of polymers or other materials with their biological effects [72]. Recent studies have also demonstrated that machine learning methods can optimize drug and dose selection, material properties, and drug and material integration configurations from the parameter space, which is difficult to achieve with traditional methods, to design tissue engineering-related systems that can predict and achieve researchers’ expectations [73]. Another strategy to facilitate biomaterial design is to integrate high-throughput platforms and machine learning techniques. For example, it has been reported that based on a library of 2304 peptides with various structures, multiple algorithms were utilized to build classification models to link the chemical structures with the self-assembly behaviours of dipeptide hydrogels. This innovative method underscores the great potential of integrating combinatorial chemistry and machine learning techniques, speeding up the design of novel peptide structures for biomedical applications [74]. Another study has successfully developed polymer structure–cell response models via machine learning algorithms and the data generated by high-throughput experiments. The best model built by an RF algorithm showed a predictive accuracy of 80% to identify anti- or pro-inflammatory materials [75]. Machine learning methods are increasingly being applied in the field of tissue engineering therapy as the basis for how to design input parameters such as drug selection and material design.

#### Machine learning in scaffold design

Scaffold design plays a pivotal role in tissue engineering as scaffolds provide the fundamental framework for tissue regeneration. Machine learning algorithms can be used to analyse the chemical, biological, and mechanical properties of different materials and polymers, predicting how the scaffold will interact with physiological tissues and organs (immune system, surrounding tissues, etc.). It helps researchers design the best scaffolds by enhancing their mechanical properties, improving biocompatibility, cell adhesion, and biodegradability, and promoting tissue regeneration [43].

The ANN model has been developed to accurately predict the degradation rate of scaffolds prepared by freeze-drying with different concentrations of gelatin and genipin, which offers a cost-effective alternative for scaffold design [47]. The physicochemical properties of polymers also contribute to evaluating the performance of scaffolds. One study has developed classification models to predict the miscibility (immiscible class and partially miscible class) of different polymer blends [46]. There is also a report of utilizing machine learning approaches to predict cell–material interactions on scaffolds, taking four numeric features characterizing the nanofibrous scaffolds (fibre diameter, pore diameter, water contact angle, and Young’s modulus) to predict the number of cells [76]. Other research reported a machine learning-based tool named MLATE to build classification models to evaluate the interactions between cardiac tissue engineering scaffolds and cells [77]. Besides, by using images from a computer-aided design (CAD) library with 20 designed lattices, CNN models have been developed to predict the calculated properties (porosity, compression modulus, and shear modulus), thus assisting new bone formation [78].

**Figure 5:**
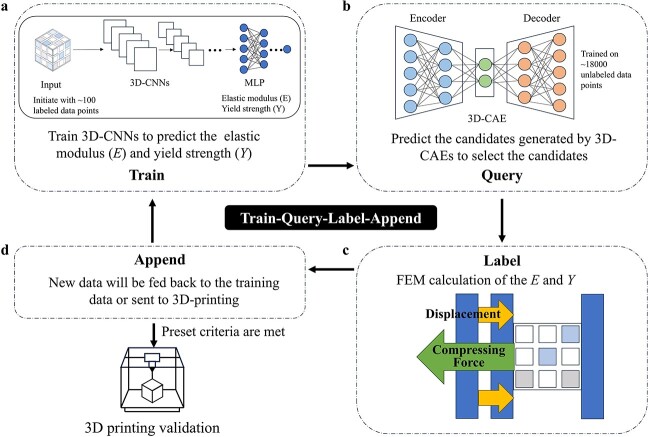
Workflow of the GAD-MALL [58]. (**a**) Train. 3D-CNNs were developed with ~100 data points to predict the elastic modules (*E*) and yield strength (*Y*). MLP, Multilayer perceptron. (**b**) Query. A 3D-CAE was trained with ~18 000 unlabelled data points to generate the scaffolds. The generated scaffolds were selected by predicted *E* and *Y* values. (**c**) Label. *E* and *Y* values of scaffold candidates were calculated by the finite element method (FEM). (**d**) Append. New scaffolds with labelled *E* and *Y* values were added to the training data. If the preset criteria are met, the active learning will stop and the chosen scaffold will be validated by 3D-printing

Recently, a workflow named generative architecture design–multi-objective active learning loop (GAD-MALL) has been reported to optimize orthopaedic implant design [58]. In this research, a 3D convolutional autoencoder (3D-CAE) trained on the unlabelled dataset consisting of about 18 000 data points, was used for candidate generation. 3D-CNNs trained on labelled data (calculated by the finite element method), were used for property prediction. As shown in Figure 5, the AL cycle started with training the 3D-CNNs based on <100 data points to predict the elastic module (*E*) and the yield module (*Y*) (train). Then, for each AL iteration, the 3D-CAE was used to generate 2000 data points with unknown properties, whose *E* and *Y* values were predicted by the 3D-CNNs to select the qualified scaffolds (query). The strain–stress curves of the selected scaffolds were further computed by the finite element method (label). The newly labelled data were subsequently fed back to the training dataset (append) to retrain the 3D-CNNs for the following active learning round. The workflow will stop when all the preset criteria are met or the learning process shows no further progress. Following this GAD-MALL workflow, high-*Y* scaffolds with *E* = 2500 MPa (termed the ‘treasure’ scaffold) were found in the third and fifth AL loop, respectively, while high-*Y* scaffolds with *E* = 5000 MPa (termed the ‘treasure’ scaffold) were found in fifth and sixth AL loop, respectively. These ‘treasure’ scaffolds significantly surpassed the ‘golden criteria’ (the uniform-designed scaffolds). Besides, compared with the baseline random search and BO, GAD-MALL showed a clear upward trend of mechanical strength improvement. The 3D printing results of designed scaffolds demonstrated that the *Y* values of the machine learning-designed scaffolds were significantly higher than that of the uniform design. This GAD-MALL workflow can also be performed without the prior data points in the target range by design, which successfully discovered treasure scaffolds with *E* = 1000 MPa. This data-efficient, intelligent method can be readily adopted in wide-ranging architected materials applications, quickly designing architected materials with various application requirements.

### Machine learning in 3D printing

3D printing is one of the most widely used tissue engineering technologies combined with machine learning. Biological 3D printing is a rapid prototyping technology based on 3D models, through the method of software layering discrete and numerical control molding, positioning, and assembling biological materials that can contain cells, so as to manufacture artificial implant scaffolds, tissues, and organs. Since the body structure and pathological condition of each patient are different, 3D bioprinting technology that is fast, accurate, personalized, differentiated, and suitable for manufacturing complex structural entities can be combined with biological materials. According to the anatomical characteristics and treatment needs of patients, medical products such as implanted scaffolds suitable for patients are manufactured using cell culture and software-assisted technology, providing new treatment technologies for personalized medicine and precision medicine [79,80]. However, the ultimate problem in bioprinting is how to find the best design and printing parameters to achieve the target geometry, mechanical, and biological functions. Both bioprinting and machine learning are key products of the fourth industrial revolution; the integration and collaboration of machine learning will help revolutionize the way bioprinting is researched and applied in clinical settings. Machine learning is expected to be quantified by objective indicators such as cell viability, biological function, printing cost, and printing duration to assist in screening the best printing device settings, which can greatly reduce the number of bioprinting trials and accelerate the process of research discovery and printing of medical products [81]. Successful bioprinting involves three steps: pre-printing, actual printing, and post-printing [82]. Machine learning can be applied to the above three stages to improve the fidelity of complex prints.

The pre-printing stage is related to the characteristics of structure, so the design and construction of suitable printing conditions can help to achieve anatomically perfect tissue models. Identifying the right bio-ink is critical and should ensure that the printability of the bio-ink is optimized while maintaining the viability and function of the cells throughout the bioprinting process. The printability of bio-inks is the ability to form pre-designed 3D structures with good shape fidelity and integrity [83,84]. Printability directly affects the mechanical properties and biological functions of scaffolds, mainly including internal factors (composition and concentration of bio-ink, etc.) and external factors (printing parameters, nozzle characteristics, printing temperature, and cross-linking conditions, etc.). Biocompatibility is also a major factor in designing bio-inks to mimic the natural environment’s ability for cells to maintain proliferation and migration [85], and researchers should ensure that a balance is struck between printability and maintaining cell viability. Researchers prepared several bio-inks using three natural collagens and meta-collagens, hyaluronic acid, and fibrin, and evaluated the printability of bio-inks using multiple regression methods, finding that high shape-fidelity requires high-frequency-dependent storage, and extrusion requires low shear stress [86]. Researchers used RF, k-nearest neighbour, ridge regression, and neural networks to determine cell viability in bio-inks for stereolithograph-based 3D bioprinting, Finally, the mechanism of influence of parameters such as ultraviolet intensity and exposure time on cell activity in bio-inks was determined [87].

It is crucial to determine the appropriate biological 3D printing parameters in the actual printing stage, and constantly changing and trying print parameters will consume a lot of time and energy and cause waste of raw materials. Combined with the powerful algorithm capability of machine learning, a suitable combination of printing parameters can be provided after model training [48]. Printing speed, layer thickness, and printing temperature are important process parameters in bioprinting that directly affect the surface quality, accuracy, strength, hardness, and toughness of printed products. Researchers used a layered machine learning framework and introduced a support vector machine algorithm to determine the optimal printing parameters (printing speed, bio-ink concentration, nozzle diameter, etc.) of a 3D scaffold composed of alginate brine gel [88]. Other researchers trained a machine learning regression model to study parameters such as cell viability and extrusion pressure in bio-inks containing alginate and gelatin. The regression model was able to predict the printing effect observed in the literature and proved the effectiveness of machine learning in bioprinting design [89]. Fully connected neural networks have also been shown to optimize parameters of inkjet bioprinting to improve printing accuracy and stability [60], and BO algorithms have been shown to optimize printing parameters of extrusion bioprinting and enhance printability while reducing the number of tests [90].

The post-printing stage is the key stage in the whole bioprinting process to determine the quality of the printed result. Machine learning promises to achieve high-quality results by integrating cellular knowledge, bioprinting, and bioprinting properties to efficiently remove auxiliary scaffolders, sanding and polishing, assembly, and surface treatment.

#### Machine learning in personalized tissue engineering design

Personalized tissue engineering is a groundbreaking approach that aims to customize tissue structures to meet the unique needs of individual patients based on their specific genetic factors, life circumstances, and disease conditions. Personalized tissue engineering can optimize the functional integration of engineered tissue with a patient’s own biological system to improve therapeutic effects and decrease the risk of implant rejection. Machine learning algorithms can analyse complex biological and clinical data (genetic data, electronic medical records, imaging data, etc.) of individual patients to predict how different scaffold materials, growth factors, and other treatment options will interact with the patient’s specific tissue and cell environment, to predict potential adverse reactions and treatment outcomes in advance.

The potential of machine learning in decision-making has already been explored by a study that took canine carotid tissue engineering as an example [91]. In this study, the tissue engineering strategies were processed into 35 input features to develop a binary classification model, and the ANN models showed accuracies of >90% to predict whether the outcomes of different vascular tissue engineering strategies are successful, indicating the great potential of machine learning in tissue engineering decision support. Although such studies are not widely reported currently, it is believed that deeper integration between machine learning and personalized tissue engineering will bring new possibilities for tailoring medical treatments to individual patients.

### Challenges and prospects

#### Challenges and strategies

Although machine learning shows great application prospects and development potential in the field of tissue engineering, the combination of the two is still in its infancy, and many key challenges need to be addressed in order to successfully implement computer-driven tissue engineering strategies.

The first major challenge is the data challenge. The computer modelling process relies on reliable data, and large amounts of data are needed to train the model (at least hundreds or thousands of data points) in order to achieve high accuracy. However, due to the lack of specialized search engines and data-extraction libraries, data acquisition and analysis will be complicated and time-consuming, and the heterogeneity of data formats, privacy, and ownership issues seriously hinder data sharing. Active advocacy by regulators could help address such issues, such as the knowledge-aided assessment and structured applications (KASA) promoted by the U.S. Food and Drug Administration (FDA) to facilitate structured data sharing. Data-sharing mechanisms, such as federated learning strategies, promise to provide rich and reliable data sets [92]. The development of standards for material testing, data collection, and preprocessing will also facilitate broader data sharing. Collaboration between researchers in machine learning and tissue engineering will accelerate disciplinary exchange and convergence and enable shared data integration. Another approach is to use advanced machine learning techniques and strategies to deal with tasks in a low-data regime. Besides the active learning we have introduced above, self-supervised learning can harness vast amounts of unlabelled data when it acts as its own label to mitigate the labour-intensive process of manual labelling. The self-supervised learning large model can serve as the start point of downstream tasks with small datasets. This strategy leverages the knowledge from a large previous model, often achieving state-of-the-art model performance [93].

The second major challenge is the interpretability of the results. Comprehensibility, interpretability, and rigorous verification are the characteristics of practical computational models. While capable of providing strong predictive accuracy, highly complex machine learning models have long been considered ‘black boxes’ where it is difficult to interpret the underlying relationships in the data, which will cause mistrust among healthcare practitioners and regulators. To eliminate the ‘black box effect’, machine learning should first ensure data accuracy, because the quality of the algorithm output is mainly determined by the input data [94]. Transparency is the key factor, e.g. models can output relationships between discovered data features and generate graphs that outline the decision-making process. Machine learning education for medical researchers is critical if machine learning techniques are to be used to design and create emerging tissue engineering strategies. Familiarity and mastery of machine learning will encourage further integration of machine learning into tissue engineering, while also increasing familiarity with interpretability and bias-related issues.

#### Prospects

In our previous review, we summarized various machine learning applications in biomaterial and scaffold design. Besides the mostly common approaches, such as developing classification and regression predictive models, several advanced machine learning technologies have not been fully exploited by tissue engineering researchers but have great potential to further advance the development of tissue engineering. For instance, recent advancements in generative machine learning models have allowed the creation of biomaterials from scratch [95]. By further combining with machine learning-based MOO, multiple properties can be optimized concurrently while generating the material’s properties [96,97], which is far more efficient than traditional laboratory trials. AL can enable tissue engineering studies to start with just a few labelled data points and autonomously select the next batch for experiments, which lowers the data volume required to perform machine learning modelling [58]. Especially, integrating AL techniques with the automated laboratory will constitute the self-driving laboratory, which is considered the potential next-generation technology for scientific experimentation [56–58]. Deeper integration between automated laboratory and machine learning techniques promises to harness the full potential of both, which may revolutionize biomaterial research and development, drastically reducing the time and resources required.

Recently, transformer-based large language models (LLMs), such as BERT [98] and GPT [99] are pushing the boundaries of what machines can understand and create. Leveraging prior knowledge on large datasets instead of building a model from scratch can offer a more useful and efficient approach to handling various tasks [100,101]. As far as we know, applications based on LLMs have not been widely reported in tissue engineering. Inspired by the outcomes already achieved by other fields, some potential applications can be listed for leveraging LLMs in tissue engineering studies. Thanks to the extremely powerful text processing of LLMs such as ChatGPT, researchers can utilize prompt engineering to interact with the model to achieve their goals, such as data mining and system literature review [102,103], as well as coding automatically [104,105]. For example, an AI system (Coscientist) driven by GPT-4 has successfully automated the design and execution of protocols for Suzuki–Miyaura and Sonogashira coupling reactions by incorporating internet and document search, code execution, and experimental automation [105]. This research also enlightens us to fine-tune LLMs with task-specific data when applying them in tissue engineering to achieve better performance. In conclusion, the collaboration between researchers and LLMs promises to drive significant advancements in the field, such as more efficient data driven experiments, ultimately benefiting a wide range of biomedical applications.

## Conclusions

The convergence of machine learning and tissue engineering is expected to open up a new era of innovation and potential. As explored in this review, we have witnessed various applications that machine learning brings to tissue engineering, from predicting biomaterial properties to optimizing scaffold designs, etc. This kind of cross-disciplinary collaboration promotes collaboration between domain experts, data scientists, and engineers, empowering them to tackle complex challenges more efficiently. Despite its vast potential, the integration of machine learning and tissue engineering is still in its infancy and faces inherent challenges. Problems like data quality, interpretability of complex models, and ethical considerations related to machine learning decision-making are among the concerns. In conclusion, while we are still in the initial stage of fully realizing the synergistic potential of machine learning in tissue engineering, it is evident that this fusion promises transformative advancements. By continually refining the methodologies and fostering interdisciplinary collaboration, the combined power of machine learning and tissue engineering will undoubtedly facilitate unprecedented innovations and possibilities in regenerative medicine.

## Abbreviations

AI: Artificial intelligence; AL: Active learning; ANN: Artificial neural networks; BO: Bayesian optimization; CNN: Convolutional neural networks; 3D-CAE: 3D Convolutional autoencoder; 3D-CNN: 3D Convolutional neural networks; CAD: computer-aided design; GAD-MALL: Generative architecture design–multi-objective active learning loop; LLM: Large language model; MOO: Multi-objective optimization; RF: Random forests; FDA: U.S. Food and Drug Administration; KASA: knowledge-aided assessment and structured applications.
